# Epidural spinal angiolipoma: a case series

**DOI:** 10.1186/s13104-017-2432-0

**Published:** 2017-03-20

**Authors:** Fei Fei Wang, Song Wang, Wen Hua Xue, Jing Liang Cheng

**Affiliations:** Department of MRI, The First Affiliated Hospital of Zhengzhou University, 1 Jian She E Road, Zhengzhou, 450052 China

**Keywords:** Spinal angiolipomas, Magnetic resonance imaging, Diffusion weight imaging

## Abstract

**Background:**

Spinal angiolipomas (SAL) are rare benign tumors, commonly presenting with slow progressive spinal cord compression. There are only about 170 cases identified so far. Magnetic resonance imaging (MRI) has become the modality of choice for SAL. The purpose of this article is to report three cases of SAL and their characteristic MRI features.

**Case presentation:**

Three cases of epidual spinal angiolipoma from ethnic Han Chinese patients are presented here, including one lumbar and two thoracic tumors. MRI imaging findings were reviewed.

**Conclusions:**

Multiple MRI technology for characterization of SAL provides useful information for differential diagnosis and therapeutic management.

## Background

Spinal angiolipomas (SAL) are rare benign tumors, accounting for about 0.14–1.2% of all spinal axis tumors and 2–3% of spinal epidural tumors [1]. They most commonly occur in mid-thoracic region, purely lumbar location is rarely reported [2, 3]. Histologically, SAL is comprised of fat tissue and abnormal vascular architecture. Depending on the predominance of the tissue components, imaging findings of the lesion varies. MRI has become the imaging modality of choice for detecting and characterizing SALs.

Here we report 3 cases of epidual spinal angiolipoma, one lumbar and two thoracic SALs, and we evaluated them by employing multiple MRI techniques including diffusion weight imaging (DWI) and dynamic contrast-enhanced (DCE) MRI.

## Case presentation

### Case 1

A 25-year-old Chinese woman with no significant medical history presented with lower back pain and left lower limb weakness and numbness for 1 year. No remarkable neurological signs were found except left ankle showed oversupination.

**Fig. 1:**
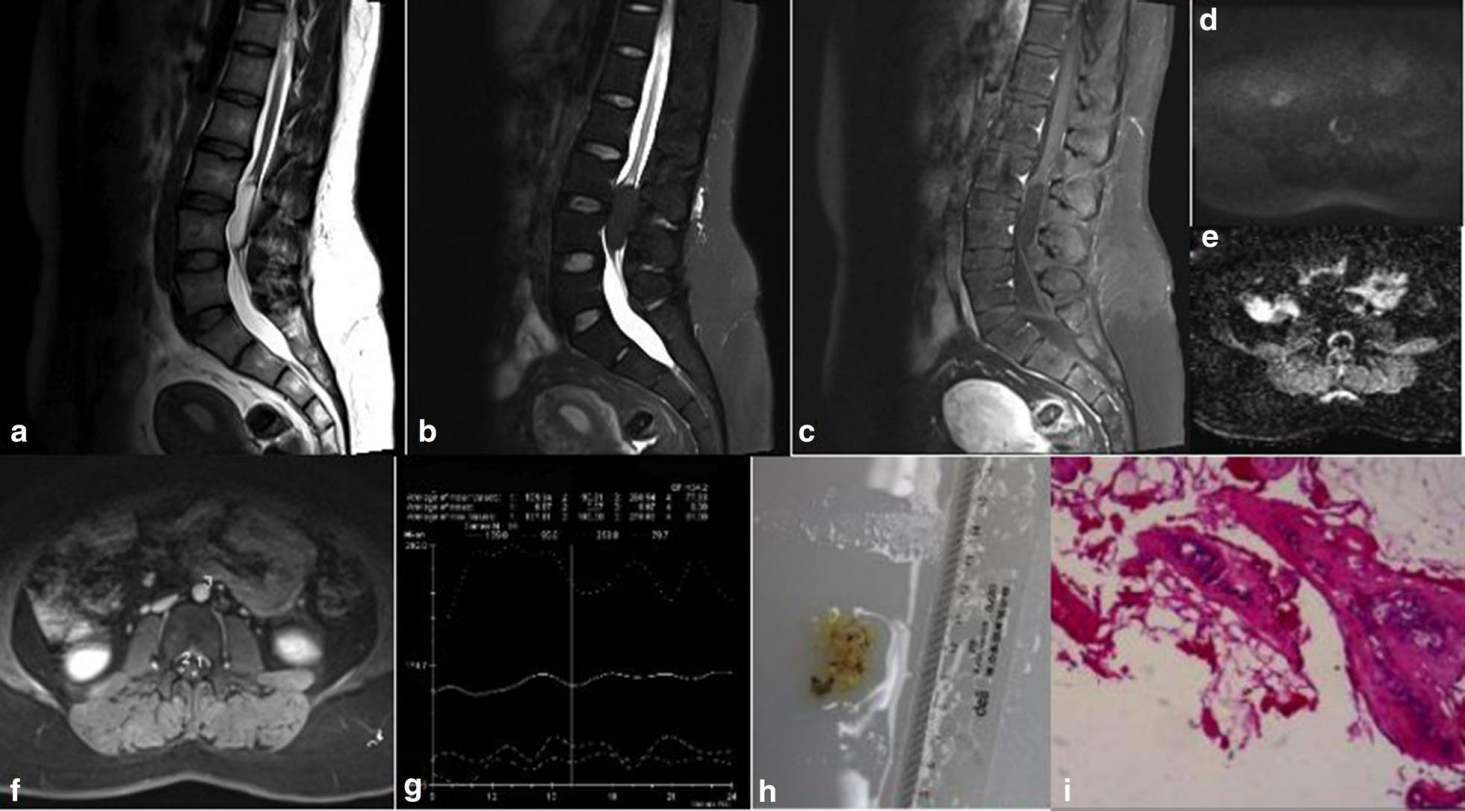
Case 1. Sagittal T2-weighted (**a**), sagittal fat suppressed T2-weighted (**b**), sagittal fat suppressed T1-weighted post-contrast (**c**) images showing an epidural mass at L3–L4 level. The mass is T2 hyperintense, hypointense in fat suppressed T2 and showed no enhancement in post-contrast images. The mass is low signal in DWI (**d**) and high signal in ADC (**e**), axial image of dynamic contrast enhanced image (**f**) showing no obvious diffusion restriction (**f**) and time-intensity curve (**g**) showed no enhancement. A *grey yellowish* mass was resected after surgery (**h**). Histological sections (**i**) shows tumor is composed of abundant vascular channel filled with red blood cells and fibrin thrombi and mature adipose tissue

MRI imaging showed an epidural mass at L3–L4 level. The mass was T2 hyperintense, hypointense in fat suppressed T2 and showed no enhancement in dynamic contrast-enhanced images (Fig. 1).

The patient underwent lumbar laminectomy and this revealed a grey yellowish tumor mass with a final pathologic measurement of 2.5 cm × 1.5 cm × 0.2 cm.

Pathological examination confirmed an angiolipoma composed of mature adult fat cells and numerous small vascular channels containing red blood cells and fibrin thrombi. Immunohistochemistry showed CK(−), CD31(+), CD34(+), S-100(+), SMA(+), Ki-67(2%+).

The patient had an uneventful recovery after the surgery, and remained asymptomatic at 2 month follow-up checkup.

### Case 2

A 77-year-old Chinese woman complained numbness of both lower extremities for 2 years, worsened for half year. The patient reported the numbness first started below umbilical level, then progressively ascended to the breast level in the recent half year. She also reported weakness and coldness of both legs, and had difficulty with balance.

Physical examination revealed reduced muscle power and tendon reflex in the lower limbs, reduced superficial sensation below breast level,bilateral lower limb Babinski(+), left Pussep(+), deep sensation impaired bilaterally, Romberg(+). The patient was unable to walk in a straight line.

**Fig. 2:**
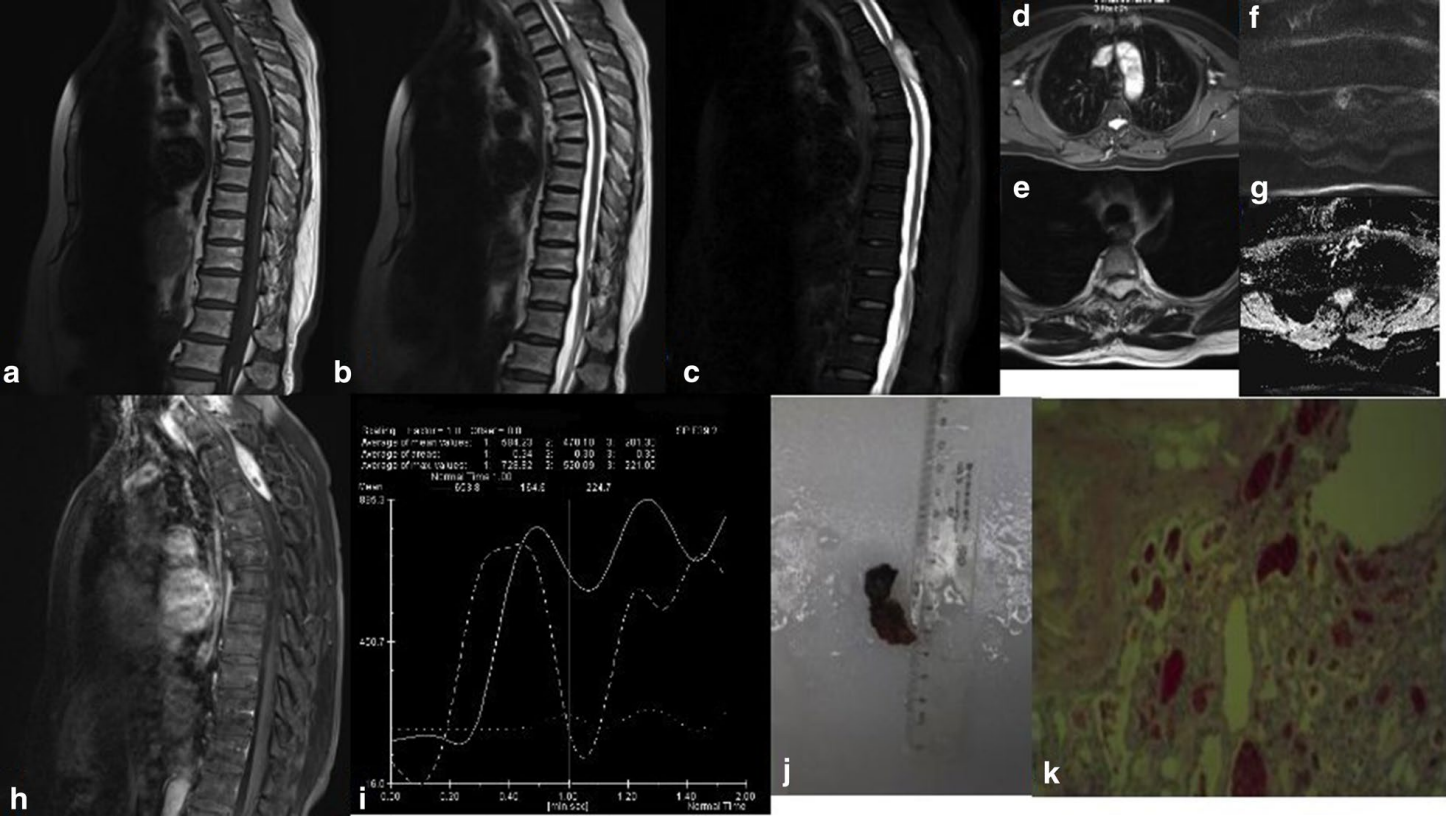
Case 2. Sagittal T1-weighted (**a**), sagittal T2-weighted (**b**), sagittal fat suppressed T2-weighted (**c**) MRI images showing a spindle shaped epidural mass extending from T2 to T4. The mass is hypointense in T1, with inhomogenous fat signals in peripheral region, hyperintense in T2 weighted imaging. The fat signals were suppressed in fat suppressed T2. Signs of thoracic and lumbar degeneration including thickening of ligamentum flavum in T10–T12 sections and disc herniation in multiple lumbar disc. Axial T2-weighted (**d**, **e**) images showed the tumor extends into bilateral neural foramen and compresses the spinal cord anteriorly. The mass is low signal in DWI (**f**) and high signal in ADC (**g**), no obvious diffusion restriction was observed. In sagittal fat suppressed T1-weighted post-contrast (**h**) image, the mass was significantly enhanced. The dynamic contrast enhanced curve (**i**) showed the rapid rising wash-in phase and wave-like washout phase without obvious decline. A *grey reddish* mass was resected after surgery (**j**). Histological sections (**k**) shows tumor is composed of abundant vascular channel filled with red blood cells and fibrin thrombi and mature adipose tissue

MRI scan demonstrated a tumor mass in the epidural space, dorsally extending from T2 to T4. The mass was hypointense in T1 weighted image, hyperintense in both T2 and fat suppressed T2 weighted images. Fat signals can be seen around the edges, showing hyperintense in both T1 and T2 weight images, but reduced signal in fat suppressed T2. DWI showed low signal of tumor but high signal in apparent diffusion coefficient (ADC) maps. Dynamic contrast-enhanced curve showed a rapid rising phase followed by a wave-like phase without obvious decay (Fig. 2).

In addition, the patient also had signs of thoracic and lumbar degeneration including thickening of ligamentum flavum in T10–T12 sections and disc herniation in multiple lumbar disc. A hemangioma at S1 level was also coexisted in this patient.

A grey reddish tumor mass that was 4 cm × 2 cm × 1 cm in size was surgically resected. Histopathological evaluation confirmed a highly vascularized angiolipoma.

The patient had an unremarkable post-surgery recovery, with relieved symptoms at 2 month follow-up checkup.

### Case 3

A 45-year-old Chinese woman presented with bilateral lower extremity numbness and stiffness for 5 months and progressively worsened 2 months prior to admission. Past medical history revealed lower back pain first appeared without obvious incentives about 10 years ago. The patient also referred right knee joint pain with bilateral lower limb weakness resulting in unstable walking 5 months ago. Physical examination showed tendon hyperreflexia and reduced power in lower extremities with bilateral Babinski signs.

**Fig. 3:**
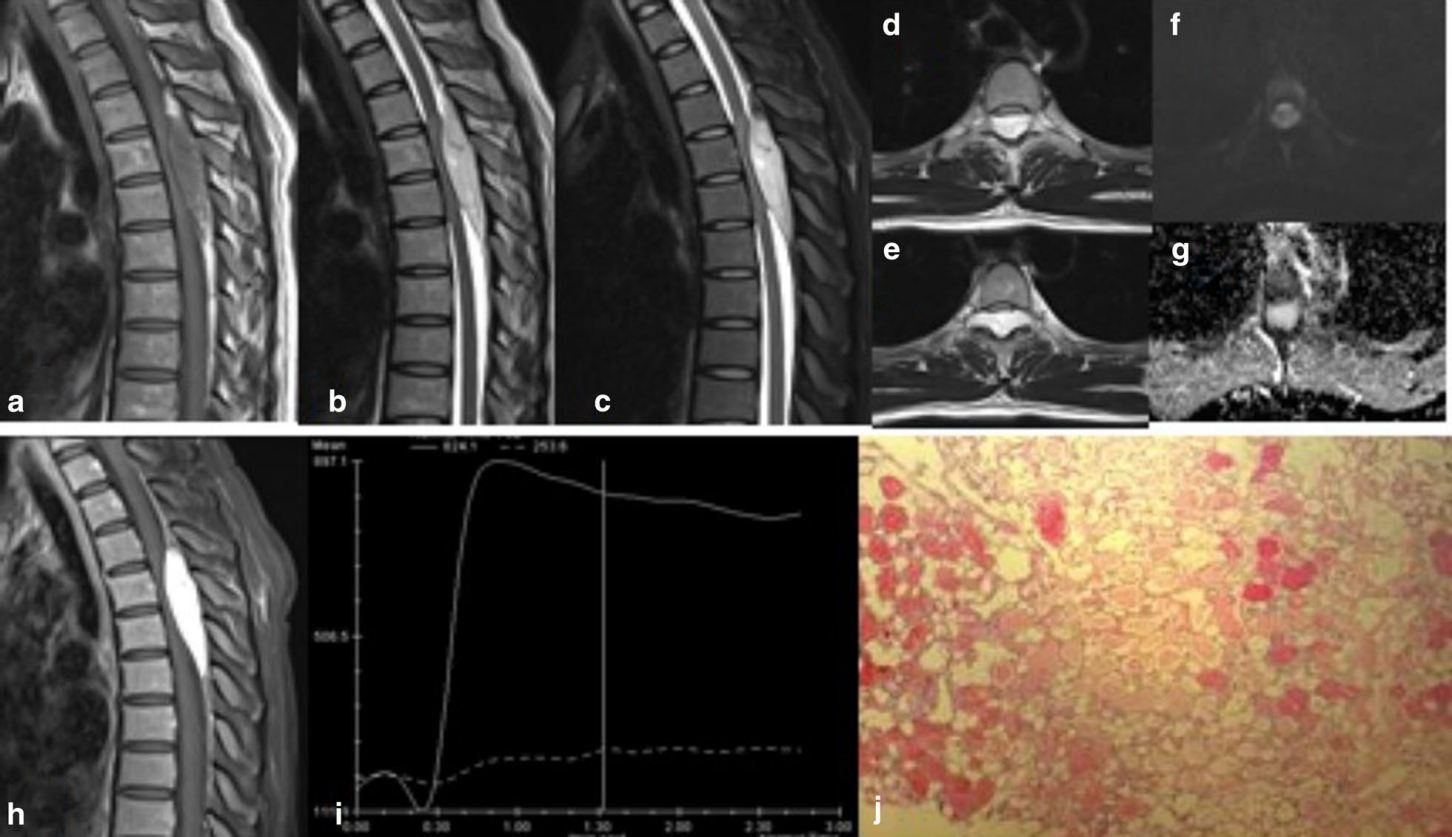
Case 3. Sagittal T1-weighted (**a**), sagittal T2-weighted (**b**), sagittal fat suppressed T2-weighted (**c**) MRI images showing a spindle shaped epidural mass extending from T4 to T6. The mass is hypointense in T1, with inhomogenous fat signals in both ends, hyperintense in T2 weighted imaging. The fat signals were suppressed in fat suppressed T2. Thin-strips or spots of vessels can be seen inside the tumor. Axial T2-weighted (**d**, **e**) images showed the tumor extends into bilateral neural foramen and compresses the spinal cord anteriorly. The mass is low signal in DWI (**f**) and high signal in ADC (**g**), no obvious diffusion restriction was observed. ADC value is 1.971 × 10^−3^mm^2^/s. In sagittal fat suppressed T1-weighted post-contrast (**h**) image, the mass is significantly and homogenously enhanced. The dynamic contrast enhanced curve (**i**) showed the rapid rising and slow decline phases, indicating the vascular component is predominating. Histological sections (**j**) shows tumor is composed of abundant vascular channel filled with red blood cells and fibrin thrombi and mature adipose tissue

A MRI scan revealed a spindle-shaped posterior epidural mass between T4 and T6, compressing the thoracic cord anteriorly. The mass was slightly hypointense on T1-weighted images, with inhomogeneous signal around the margins, and small area of fat signal in upper margin. It was hyperintense on T2-weighted images, and the fat signal in upper margin was repressed on fat suppressed T2 weighted images. On the axial T2 weighted images, the lesion was shown to extend into the adjacent intervertebral foramen. The tumor showed low signal in DWI but high signal in ADC maps. Dynamic contrast-enhanced curve showed a rapid rising phase followed by a slow decay phase indicating vascular component is predominating (Fig. 3).

The patient was subjected to laminectomy under general anesthesia. During the operation, a yellowish, highly vascularized mass with clear margin was found in the posterior epidural space, and was totally excised.

Pathological examination disclosed an angiolipoma composed of mature adult fat cells and numerous small vascular channels containing red blood cells and fibrin thrombi. Immunohistochemistry revealed CD34(+), S-100(+), HMB45(+), Ki-67(5%+).

The patient had an uneventful recovery after the surgery, and regained normal neurological functions after the follow-up period of 10 months.

## Discussion

First reported in 1890 by Berenbruch [4] spinal epidural angiolipomas are rare benign tumors composed of mature adipocytes and abnormal vasculature in varied proportions. There were 177 patients with spinal epidural angiolipoma identified up to June 2015, with an average age of 46 ± 16 years old and 59% of patients were females [5]. The tumors are predominantly located in thoracic spine, pure lumbar angiolipomas are rather rare with less than 20 cases ever reported [2, 3]. About 90% of lesion arises in the posterior epidural spaces, extending over several vertebral bodies [6].

The clinical symptoms of SALs are consistent with other benign, space-occupying spinal lesions that are related to spinal cord/nerve root compression, or infiltration of surrounding tissue. The most common initial symptoms include lower limb numbness or paraesthesia, leg weakness and back pain. It can evolve over a span of months to years, but occasionally sudden onset or worsening of neurological symptoms occurred when there is a rapid increase in tumor size due to intratumoral thrombosis, haemorrhage [7, 8].

Etiology and pathogenesis of spinal angiolipoma remain largely unknown. It was proposed that an insufficiency of blood supply in thoracic spine contributes to the thoracic predominance of SAL [9]. In addition, it was reported that coexistence of vertebral hemangiomas is common in SAL patients, 23.8% cases (5/21) in one study [10] and one case (#2) in our report, indicating an abnormal vessel formation and growth mechanism. In fact, some authors consider SAL in the middle of a disease spectrum with lipoma at one end and hemangioma in the other end [16]. Several predisposing factors were also suggested to be associated with SAL, such as weight gaining or hormonal changes in pregnancy [11] and elevated body mass index [10], however, systematic studies on the etiology of SALS are still lacking.

The first pathological report describing anatomopathological composition of SAL was done by Howard et al. [12]. Under gross examination, the lesion of SAL is grey reddish in color, soft texture, with none or incomplete encapsulation, easy to separate from peripheral tissue. The tumor grows in epidural space along the longitudinal axis, normally in spindle shape. It can spread along the intervertebral foramen and presented as dumbbell shaped lesion. Traditionally, SALs are categorized into two types: noninfiltrating and infiltrating SAL. The noninfiltrating type is more common, usually well encapsulated and demarcated, while the infiltrating types are rare, partially or entirely unencapsulated, ill defined, and commonly infiltrate the surrounding tissues especially the bone [13], therefore should be considered and treated differently [14]. However, in recent years, some researchers indicated most patients achieved good recovery and there is no difference in the outcomes of the two types [15]. All 3 cases in this report can be categorized into the none-infiltrating type as they showed clear demarcation and no invasion to the bone.

Histological, SAL is a lesion with two distinct components: mature adipose tissue and proliferating abnormal blood vessels including capillary, sinusoidal, venous or arterial vascular elements [13]. The ratio of fat to vessels is variable [16], and atypia, pleomorphism, and mitotic figures of both adipose and angiomatous component were never observed [17]. Immunohistochemical assays were performed in 2 cases, and we observed a positive stain for CD31, S-100, SMA, but low proliferation rate was found with Ki-67, which is in agreement with others’ findings [18, 19].

Currently, MRI is the best imaging modality of choice in the diagnosis of SAL. The common features of SALs in MRI are presented as a spindle shaped lesion with both ends pen-like, usually located in the posterior epidural space extending longitudinally along the spine. The normal dura displayed as a slit-like low signal on T2-weighted imaging between the tumor and the spinal cord, and the subarachnoid space was narrowed and the spinal cord displaced. Occasionally the lesion may extend into the adjacent intervertebral forame with foraminal widening. The MR signal of SAL is comprised of two components: adipose tissue and vascular architecture. The fatty content was hyperintense on both T1- and T2-weighted images, hypointense on fat-suppressed images. The vascular component was hypointense on T1-weighted and hyperintense on T2-weighted, and showed intense enhancement in post-contrast images. Depending on its composite ratio of fat tissue versus vascular component, the lesion displayed as inhomogenous in pre-contrast images. Vascular flow voids sign is rarely seen on the MR images, as there is usually lack of well-developed arterioles inside the tumor.

In this case report, the MRI images of case 2 and case 3 demonstrated features of vascular component dominating SAL, as hypointense in T1, hypertintense in T2 and remarkable enhancement post-contrast. This type of SAL can be differentiated from other vascular originated tumors like hemangioma in that the fat signals from SAL are part of the tumor itself while in hemangioma the surrounding fat tissue clearly delineated from the tumor. Vascular void sign can also used to distinguish from arteriovenous malformation which is rarely seen in SALs. However, in this report one strip of vascular void signal was shown in case 2.

Case 1 can be easily confused with lipoma as it showed hyperintense in T2, reduced signal in fat suppressed T2, and no contrast enhancement. In this particular case, contrary to previous report by Hu et al. [6] fat suppression is not very helpful to reveal any vascular component. SAL was finally confirmed by histology and immunochemistry.

Dynamic contrast-enhanced MRI (DCE-MRI) was performed to assess the tumor vascular characteristics. The three SAL cases showed distinct shape in the time-intensity curve. No enhancement was observed in case one, while there were initial onsets of wash-in phase observed in both case two and three, followed by a wave-like washout phase in case two, and slow decaying washout phase in case three.

Diffusion-weighted imaging (DWI) is now widely appreciated as an indispensable tool in the examination of the central nervous system (CNS), especially for detection of acute ischaemic stroke and for characterization and differentiation of CNS tumors. In this study, all three cases of SAL showed low signal on DWI map, but high signal on ADC diagram, with no obvious diffusion restriction. A large scale of study would be helpful in order to assess the utility of DWI in the diagnosis of SAL.

SALs are treated exclusively by surgical removal of the lesion, gross total resection for noninfiltrating SALs and sub-total excision for infiltrating ones. The outcomes of surgery are good and recurrence is unusual in SALs.

## Conclusions

In summary, we reported three cases of SAL with the emphasis on their MRI features. MRI findings indicate that one case is fat tissue predominant and the other two cases are vascular component predominant. By employing multiple MRI technology, better characterization of SAL can be obtained in regard to the composition and infiltration of the tumor. In spite of the ever-growing acuity of MRI imaging, diagnosis of SAL should be made in combination with clinical, radiological and pathological examination findings.

## Abbreviations

SAL: spinal angiolipomas
MRI: magnetic resonance imaging
DWI: diffusion weight imaging
DCE: dynamic contrast-enhanced
ADC: apparent diffusion coefficient
